# Diagnostic value of high-frequency ultrasound in preoperative evaluation of benign and malignant skin tumors

**DOI:** 10.3389/fonc.2026.1819735

**Published:** 2026-06-10

**Authors:** Yang Zhao, Yu-Jing Zhou, Dan-Hua Li, Ting Liang, Yu-Jing Zhao, Dan-Dan Shan, Chuan Qin

**Affiliations:** 1 Department of Medical Ultrasound, Jinshan Hospital of Fudan University, Shanghai, China; 2 Department of Radiology, Shanghai Skin Disease Hospital of Tongji University, Shanghai, China; 3 Department of Medical Ultrasound, Shanghai Tenth People’s Hospital, School of Medicine, Tongji University, Shanghai, China; 4 Department of Medical Ultrasound, Shanghai Skin Disease Hospital of Tongji University, Shanghai, China

**Keywords:** benign tumor, high-frequency ultrasound, malignancy, nomogram, skin tumor

## Abstract

**Objective:**

To develop a High-frequency ultrasound (HFUS)-based predictive nomogram integrating clinical and ultrasound features for distinguishing malignant from benign skin tumors.

**Methods:**

This retrospective study enrolled 185 patients who underwent preoperative HFUS examination for skin tumors. Patients were randomly divided into training (n=130; 73 malignant, 57 benign) and validation (n=55; 31 malignant, 24 benign) cohorts. Clinical characteristics and ultrasound features were systematically analyzed. Univariate and multivariate logistic regression analyses were used to identify independent predictors of malignancy, which were incorporated into a nomogram. Model performance was evaluated using receiver operating characteristic (ROC) curves, calibration plots, and decision curve analysis (DCA).

**Results:**

Malignant tumors demonstrated significantly greater multilayer involvement, irregular margins, heterogeneous echogenicity, raised surface morphology, and abundant intralesional vascularity compared to benign tumors (all P<0.001). Multivariate analysis identified five independent predictors of malignancy: advanced age (OR = 1.01, 95% CI: 1.00 - 1.01), multilayer involvement (OR = 1.19, 95% CI: 1.12 - 1.26), unclear margins (OR = 1.36, 95% CI: 1.21 - 1.54), raised surface morphology (OR = 1.10, 95% CI: 1.01 - 1.21), and heterogeneous echogenicity (OR = 1.09, 95% CI: 0.99 - 1.21). The nomogram achieved area under the curve (AUC) values of 0.98 (95% CI 0.95-1.00) in the training cohort and 0.98 (95% CI 0.95-1.00) in the validation cohort.

**Conclusions:**

HFUS provides valuable information for preoperative evaluation of skin tumors. The nomogram demonstrates good diagnostic performance and clinical utility for distinguishing malignant from benign skin tumors.

## Introduction

Skin tumors are among the most common neoplastic diseases in humans (1). Owing to diverse cellular origins and complex histopathological compositions, skin tumors exhibit heterogeneity in biological behavior and clinical presentation. Clinically, skin tumors can be broadly classified into benign and malignant types (2). While benign skin tumors are generally slow-growing and localized, malignant skin tumors-such as basal cell carcinoma, squamous cell carcinoma, and malignant melanoma-are characterized by invasive growth, which increases morbidity and mortality significantly (3). Given this clinical heterogeneity, accurate preoperative differentiation between benign and malignant skin tumors is essential to guide appropriate management.

Early and precise preoperative evaluation plays a critical role in the management of skin tumors (4). Traditionally, clinical examination methods such as visual inspection supplemented by dermoscopy have been used for preliminary assessment (5). However, this visual abasement is limited by subjectivity and often insufficient for evaluating tumor depth, internal structure, and relationships with surrounding tissues (6). Histopathological examination remains the gold standard for diagnosis. However, it is invasive and can cause scarring, bleeding, or cosmetic damage (7, 8). These limitations highlight the need for reliable, noninvasive imaging tools capable of objectively evaluating tumor morphology and depth prior to surgery.

Conventional two-dimensional ultrasound is a noninvasive, real-time, and cost-effective modality without ionizing radiation (9). However, due to the limited spatial resolution, its diagnostic accuracy in distinguishing malignant from benign skin tumors remains suboptimal (10). High-frequency ultrasound (HFUS) is ultrasound operating at frequencies above 20 MHz (11). HFUS offers superior spatial resolution, enabling detailed visualization of skin layers and precise measurement of tumor thickness, which is well suited for preoperative assessment of skin tumors (12). However, the extent to which HFUS-derived features can be systematically integrated into a quantitative predictive model for preoperative risk stratification has not been fully explored.

Despite its increasing clinical use, the diagnostic value of HFUS in differentiating malignant from benign skin tumors and in guiding preoperative evaluation has not been fully established. Therefore, the present study aims to investigate the role of HFUS in the preoperative assessment of skin tumors by analyzing the imaging features and diagnostic performance. By comparing ultrasound findings with postoperative histopathological results, this study seeks to clarify the clinical utility of HFUS in improving diagnostic accuracy, optimizing surgical planning, and ultimately enhancing patient outcomes.

## Materials and methods

### Ethics

This study was conducted in accordance with the Declaration of Helsinki. This retrospective cohort study was clinically and ethically approved by the Ethics Committee of Shanghai Skin Disease Hospital of Tongji University (Date: 2023.05.25, Number: 2023-10). Due to the retrospective nature of the study, the requirement for informed consent was waived.

### Study design and patients

This retrospective study analyzed patients with skin tumors who underwent preoperative ultrasound examination followed by surgical excision between November 2021 and December 2024 at Jinshan Hospital, Fudan University, and Shanghai Skin Disease Hospital. All enrolled patients had complete preoperative ultrasound imaging data and definitive postoperative histopathological diagnoses.

Ultrasound images were stored in a centralized database and retrospectively reviewed. Histopathological slides were re-evaluated to confirm tumor type, and tumors were classified as benign or malignant based on pathological findings. The entire dataset was randomly divided into a training cohort and a validation cohort at a ratio of 7:3 for model development and validation. The training cohort was used exclusively for predictor identification and nomogram construction via multivariate logistic regression. The validation cohort was used to independently assess the nomogram’s predictive performance and to evaluate its generalizability, thereby reducing the risk of overfitting.

### Inclusion and exclusion criteria

Inclusion criteria were as follows: Patients with clinically suspected skin tumors who underwent preoperative ultrasound examination; Surgical excision performed within a defined period after ultrasound evaluation; Availability of complete ultrasound imaging data and clinicopathological information. Exclusion criteria included: Poor-quality ultrasound images unsuitable for analysis; Prior biopsy, surgery, or treatment to the tumor before ultrasound examination; Incomplete clinical or pathological data.

### Ultrasound examination protocol

Ultrasound examinations were performed using high-resolution ultrasound systems equipped with high-frequency linear-array transducers. The equipment used for the HFUS examination of skin lesions was as follows: (a) an HFUS linear-array transducer (4–20 MHz, Aix-en-Provence, France, Aixplorer; Super Sonic Imagine); (b) an HFUS linear-array transducer (10–22 MHz, Genova, Italy, My LabTM class C; Esaote SpA); (c) an HFUS linear-array transducer (22–38 MHz, Beijing, China, Paragon XHDTM class C; KOLO); and (d) ultrasound biological microscopy (UBM) equipped with a mechanically driven linear transducer (50 MHz, Tianjing, China, MD300SII; Meda Co, Ltd.). The selection of the transducer was based on the size and thickness of the lesion in order to achieve optimal imaging. Initially, a lower frequency was used to scan the lesions for their precise location and measure each lesion’s thickest part. Subsequently, an appropriate probe frequency was selected for detailed evaluation based on the thickness of the lesion. For extremely thin lesions, UBM was used for the scanning. All ultrasound scans were conducted per the established guidelines for diagnosing skin diseases.

### Image evaluation and ultrasound features

The following clinical and ultrasound variables were recorded for each tumor: Demographic and clinical data: sex, age, tumor location, and clinical diagnosis (made independently by two dermatologists using dermoscopy; discrepancies resolved by consensus); Layer involvement: epidermis; epidermis + dermis; epidermis + dermis + subcutaneous tissue; Number of involved layers: single layer, two layers, or full-thickness skin involvement; Morphology: regular, creeping, or irregular shape; Margin characteristics: smooth, spiculated/irregular, or ill-defined; Basal boundary: clear or unclear; Internal composition: cystic, solid, or mixed; Echogenicity: homogeneous or heterogeneous; Calcification: absent or present; Surface contour: flat, depressed, elevated, or completely protruding above the skin surface; Epidermal keratinization: absent, normal, or excessive; Posterior acoustic features: unchanged, attenuated, or enhanced; Vascularity: Adler grade (0–3); Histopathological diagnosis: Independently assessed by two experienced pathologists, with discrepancies resolved by consensus.

Ultrasound image analysis was independently performed by two experienced sonographers who were blinded to pathological results. Both observers underwent standardized training prior to image evaluation. Only features with interobserver agreement were included in the final analysis.

### Color doppler flow imaging

Color Doppler flow imaging was used to evaluate intralesional and perilesional vascularity. The probe was applied without compression, and the sampling box was adjusted to slightly exceed the tumor boundaries. Doppler parameters, including color gain, baseline, and pulse repetition frequency, were optimized, with the velocity scale appropriately reduced to detect low-velocity flow.

Tumor vascularity was assessed using the Adler semi-quantitative grading system, classified as follows: Grade 0: No detectable blood flow within or around the tumor;

Grade 1: Minimal flow, with 1–2 punctate or short linear vessels; Grade 2: Moderate flow, with 3–4 punctate vessels or one longer vessel extending close to or beyond the tumor radius; Grade 3: Marked vascularity, with ≥5 punctate vessels or two or more long vessels.

### Logistic regression analysis

Univariate logistic regression analysis was initially performed to identify ultrasound features associated with tumor malignancy. Variables showing statistical significance were subsequently entered into a multivariate logistic regression model to determine independent predictors of malignant skin tumors.

### Nomogram construction and validation

Independent predictors identified through multivariate logistic regression were linearly integrated to construct a nomogram for preoperative prediction of tumor malignancy.

The performance of the nomogram was assessed using receiver operating characteristic (ROC) curves and area under the curve (AUC) and calibration curves (evaluating agreement between predicted and observed outcomes). Internal validation was performed using the validation cohort.

### Clinical utility assessment

The diagnostic performance of the nomogram was compared with clinical diagnoses made by dermatologists using dermoscopy. Decision curve analysis (DCA) was applied to evaluate and compare the clinical net benefit of the nomogram and clinical assessment across a range of threshold probabilities.

### Statistical analysis

All statistical analyses were conducted using R software (Version 4.5.0). Continuous variables were reported as mean ± standard deviation (SD). The normality of data distribution was assessed using the Shapiro-Wilk test. Comparisons of continuous variables between groups were carried out using either Student’s t-test for normally distributed data or the Mann-Whitney U test for non-normally distributed data. For categorical variables, comparisons were performed using Chi-square tests, or Fisher’s exact test. Correlation analyses were carried out using either Pearson’s correlation coefficient for normally distributed variables or Spearman’s correlation coefficient for non-normally distributed or ordinal variables. All tests were two-tailed, and statistical significance was defined as p < 0.05.

## Results

### Patient characteristics

**Figure 1:**
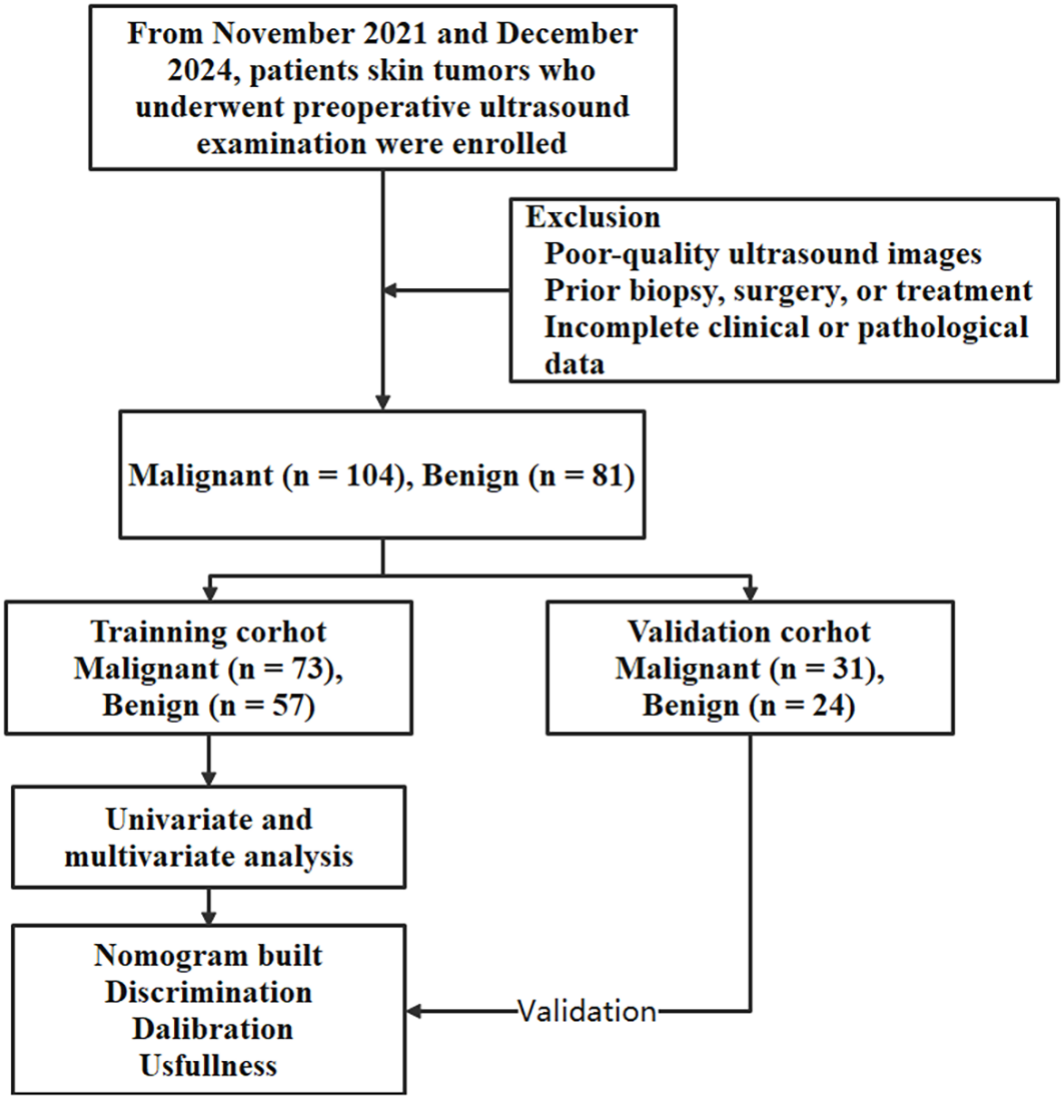
Flowchart of patient selection and study design.

A total of 185 skin tumors were included: 130 in the training cohort (57 benign, 73 malignant) and 55 in the validation cohort (24 benign, 31 malignant, **Figure 1**). Across both cohorts, sex distribution did not differ significantly between benign and malignant groups (training: P = 0.453; validation: P = 0.767). In contrast, patients with malignant tumors were significantly older than those with benign tumors in both cohorts (both P < 0.001). Regarding tumor location, malignant tumors were more frequently situated on the head and face in the training cohort (P = 0.005); this difference was not statistically significant in the validation cohort (P = 0.499), likely reflecting the smaller sample size of the latter (**Table 1**).

**Table 1:** Clinical and ultrasound characteristics of benign and malignant skin tumors in training and validation cohorts.

	Training cohort			Validation cohort		
Parameters	Benign	Malignant	P-value	Benign	Malignant	P-value
	(N = 57)	(N = 73)		(N = 24)	(N = 31)	
Gender			0.453			0.767
Female	32 (56.1%)	35 (47.9%)		9 (37.5%)	14 (45.2%)	
Male	25 (43.9%)	38 (52.1%)		15 (62.5%)	17 (54.8%)	
Age	39.7 ± 18.0	70.9 ± 15.1	<0.001	46.9 ± 20.7	73.3 ± 11.5	<0.001
Site						0.499
Head and Face	17 (29.8%)	42 (57.5%)	0.005	8 (33.3%)	15 (48.4%)	
Limb	14 (24.6%)	14 (19.2%)		6 (25.0%)	7 (22.6%)	
Trunk	26 (45.6%)	17 (23.3%)		10 (41.7%)	9 (29.0%)	
Clinical diagnosis			<0.001			<0.001
Benign	52 (91.2%)	13 (17.8%)		23 (95.8%)	12 (38.7%)	
Malignant	5 (8.8%)	60 (82.2%)		1 (4.2%)	19 (61.3%)	
Layers Involvement			<0.001			<0.001
Dermis	13 (22.8%)	0 (0%)		5 (20.8%)	0 (0%)	
Epidermis	6 (10.5%)	3 (4.1%)		4 (16.7%)	1 (3.2%)	
Epidermis and dermis	6 (10.5%)	39 (53.4%)		2 (8.3%)	15 (48.4%)	
Epidermis and dermis and subcutaneous	1 (1.8%)	31 (42.5%)		1 (4.2%)	15 (48.4%)	
Subcutaneous	31 (54.4%)	0 (0%)		12 (50.0%)	0 (0%)	
Layer levels			<0.001			<0.001
One	49 (86.0%)	3 (4.1%)		21 (87.5%)	1 (3.2%)	
Two	7 (12.3%)	39 (53.4%)		2 (8.3%)	15 (48.4%)	
Three	1 (1.8%)	31 (42.5%)		1 (4.2%)	15 (48.4%)	
Shape			<0.001			<0.001
Creeping	8 (14.0%)	9 (12.3%)		3 (12.5%)	12 (38.7%)	
Irregular	6 (10.5%)	52 (71.2%)		2 (8.3%)	13 (41.9%)	
Regular	43 (75.4%)	12 (16.4%)		19 (79.2%)	6 (19.4%)	
Edge			<0.001			<0.001
Smooth and neat	47 (82.5%)	13 (17.8%)		21 (87.5%)	0 (0%)	
Unclear	10 (17.5%)	60 (82.2%)		3 (12.5%)	31 (100%)	
Basal part			<0.001			0.001
Clear	44 (77.2%)	22 (30.1%)		18 (75.0%)	8 (25.8%)	
Unclear	13 (22.8%)	51 (69.9%)		6 (25.0%)	23 (74.2%)	
Internal characteristics			<0.001			0.007
Cystic	12 (21.1%)	0 (0%)		6 (25.0%)	0 (0%)	
Mixed	9 (15.8%)	3 (4.1%)		3 (12.5%)	2 (6.5%)	
Solid	36 (63.2%)	70 (95.9%)		15 (62.5%)	29 (93.5%)	
Calcification			<0.001			0.002
None	54 (94.7%)	36 (49.3%)		23 (95.8%)	17 (54.8%)	
Present	3 (5.3%)	37 (50.7%)		1 (4.2%)	14 (45.2%)	
Surface			<0.001			0.363
Flat	31 (54.4%)	7 (9.6%)		9 (37.5%)	7 (22.6%)	
Raised	26 (45.6%)	66 (90.4%)		15 (62.5%)	24 (77.4%)	
Epidermal keratinization			<0.001			0.007
None	52 (91.2%)	33 (45.2%)		18 (75.0%)	11 (35.5%)	
Normal keratinization	1 (1.8%)	14 (19.2%)		2 (8.3%)	2 (6.5%)	
Over keratinization	4 (7.0%)	26 (35.6%)		4 (16.7%)	18 (58.1%)	
Echo			<0.001			0.033
Non-uniform	26 (45.6%)	58 (79.5%)		11 (45.8%)	24 (77.4%)	
Uniform	31 (54.4%)	15 (20.5%)		13 (54.2%)	7 (22.6%)	
Posterior echo			<0.001			0.001
Attenuated	5 (8.8%)	17 (23.3%)		4 (16.7%)	5 (16.1%)	
Enhanced	19 (33.3%)	4 (5.5%)		10 (41.7%)	1 (3.2%)	
Unchanged	33 (57.9%)	52 (71.2%)		10 (41.7%)	25 (80.6%)	
CDFI			<0.001			<0.001
I	4 (7.0%)	3 (4.1%)		3 (12.5%)	0 (0%)	
Ii	2 (3.5%)	2 (2.7%)		4 (16.7%)	28 (90.3%)	
Iii	6 (10.5%)	67 (91.8%)		17 (70.8%)	0 (0%)	
None	45 (78.9%)	1 (1.4%)		0 (0%)	3 (9.7%)	

CDFI, Color Doppler Flow Imaging; Echo, Echogenicity; Posterior echo, Posterior Acoustic Features; SD, Standard Deviation; HFUS, High-Frequency Ultrasound

### Clinical and histopathological diagnoses

Dermoscopic clinical diagnosis demonstrated acceptable accuracy in both cohorts. In the training cohort, 91.2% of benign tumors and 82.2% of malignant tumors were correctly classified clinically. Comparable performance was observed in the validation cohort, where 95.8% of benign tumors and 61.3% of malignant tumors were correctly identified. The notably lower malignancy detection rate in the validation cohort (61.3% vs. 82.2%) suggests that clinical dermoscopic assessment alone may be insufficient for reliably identifying malignant lesions, further underscoring the need for objective imaging-based tools such as HFUS. Histopathological analysis confirmed that benign tumors comprised predominantly epidermoid cysts, lipomas, nevi, fibromas, and keloids, while malignant tumors included basal cell carcinoma, squamous cell carcinoma, melanoma, sweat gland carcinoma, trichilemmal carcinoma, and verrucous carcinoma (**Table 2**).

**Table 2:** Histopathological distribution of benign and malignant skin tumors in training and validation cohorts.

Histological diagnosis	Training cohort		Validation cohort	
	Benign (N = 57)	Malignant (N = 73)	Benign (N = 24)	Malignant (N = 31)
Epidermoid cyst	20 (35.1%)	0 (0%)	8 (33.3%)	0 (0%)
Hemangioma	2 (3.5%)	0 (0%)	2 (8.3%)	0 (0%)
Fibroma	8 (14.0%)	0 (0%)	0 (0%)	0 (0%)
Keloid	5 (8.8%)	0 (0%)	3 (12.5%)	0 (0%)
Lipoma	7 (12.3%)	0 (0%)	4 (16.7%)	0 (0%)
Nevus	13 (22.8%)	0 (0%)	4 (16.7%)	0 (0%)
Other	2 (3.5%)	4 (5.5%)	3 (12.5%)	2 (6.5%)
Basal cell carcinoma	0 (0%)	27 (37%)	0 (0%)	9 (29.1%)
Melanoma	0 (0%)	4 (5.5%)	0 (0%)	3 (9.7%)
Squamous cell carcinoma	0 (0%)	26 (35.6%)	0 (0%)	12 (38.7%)
Sweat gland carcinoma	0 (0%)	4 (5.5%)	0 (0%)	1 (3.2%)
Trichilemmal carcinoma	0 (0%)	4 (5.5%)	0 (0%)	3 (9.7%)
Verrucous carcinoma	0 (0%)	4 (5.5%)	0 (0%)	1 (3.2%)

Other, including Paget, Mucinous carcinoma, Paget, Bowen, Dermatofibrosarcoma, Paget, Keratoacanthoma, Trichoblastoma, Verruca vulgaris, Seborrheic keratosis, Seborrheic keratosis.

### Skin layer involvement

Significant differences were observed in layer involvement patterns between benign and malignant tumors in both cohorts (both P < 0.001) (**Table 1**). Benign tumors were more likely to be confined to a single skin layer, particularly the subcutaneous layer. However, malignant tumors more frequently involved multiple skin layers, especially the epidermis and dermis, or full-thickness skin involvement including subcutaneous tissue. Similarly, malignant tumors commonly affected two or three skin layers, while benign tumors were mainly limited to one layer (both P < 0.001).

### Morphological ultrasound features

Significant differences in tumor morphology were observed between benign and malignant tumors in both cohorts (**Table 1**). Malignant tumors more commonly exhibited irregular or infiltrative morphology, with spiculated or ill-defined margins and an unclear deep basal boundary (all P < 0.001 in both cohorts). Conversely, benign tumors were predominantly regular in shape, with smooth, well-defined margins and a clearly demarcated basal boundary (P < 0.001 in both cohorts).

### Internal characteristics and echogenicity

Malignant tumors were predominantly solid. However, benign tumors more frequently demonstrated cystic or mixed internal characteristics (P < 0.001 in the training cohort; P = 0.007 in the validation cohort) (**Table 1**). The presence of calcification was significantly more common in malignant tumors than in benign ones in both cohorts (P < 0.001). Malignant tumors were more commonly to show heterogeneous echogenicity, whereas benign tumors more often displayed homogeneous echotexture. 

### Surface features and epidermal changes

Malignant tumors were significantly more likely to exhibit a raised surface contour, whereas benign tumors frequently appeared flat or mildly elevated (P < 0.05 in both cohorts) (**Table 1**). In addition, epidermal keratinization, particularly over-keratinization, was significantly more common in malignant tumors than in benign ones in both cohorts (P < 0.001) (**Table 1**).

### Posterior acoustic features

Posterior acoustic features differed significantly between benign and malignant tumors (**Table 1**). Posterior acoustic enhancement was more commonly observed in benign tumors, while malignant tumors predominantly demonstrated unchanged or attenuated posterior echoes (P < 0.001).

### Color doppler flow imaging

**Figure 2:**
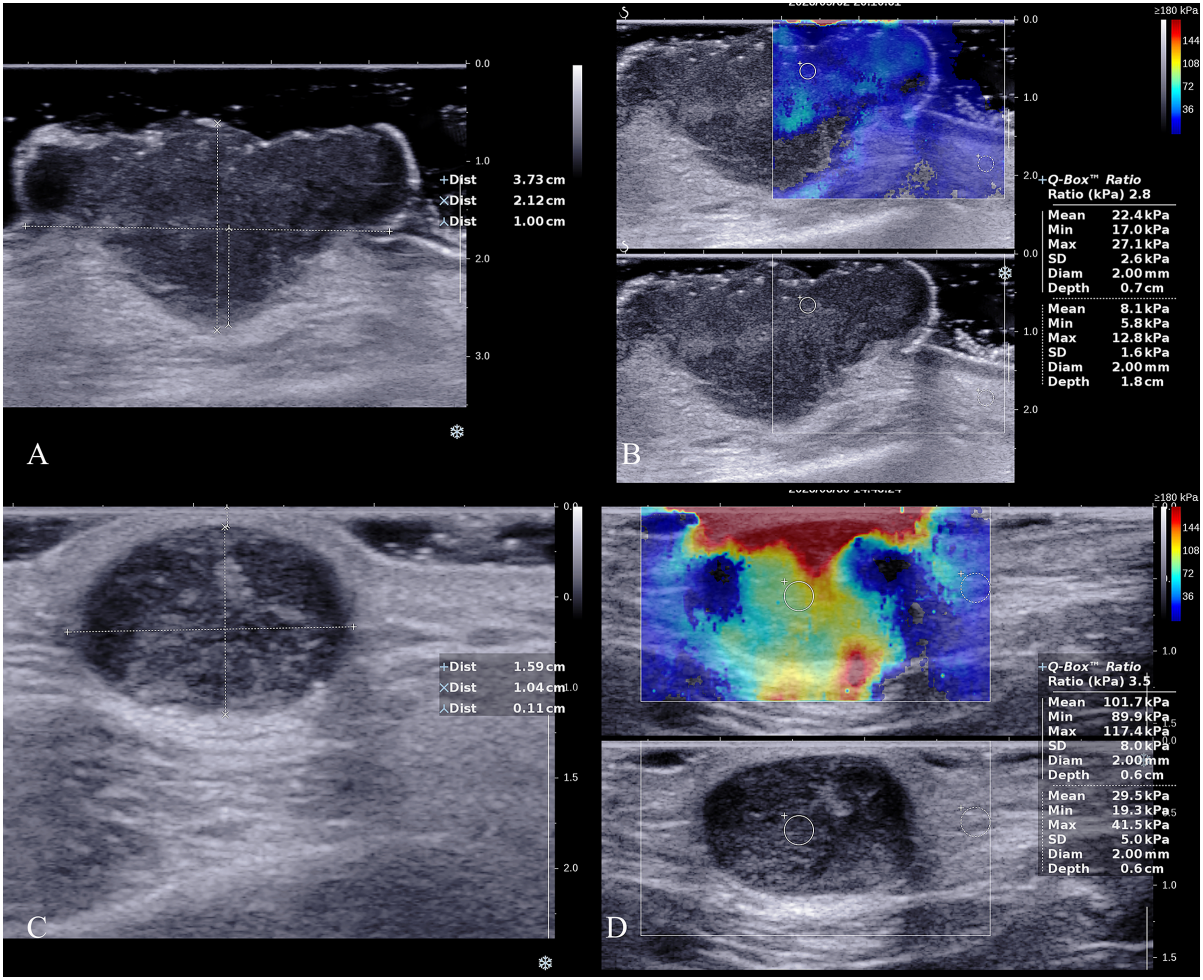
Representative high-frequency ultrasound (HFUS) and color Doppler imaging features of malignant (Squamous cell carcinoma) and benign skin tumors (Epidermoid cyst). **(A)** HFUS image demonstrating a hypoechoic tumor with multilayer involvement (epidermis, dermis, and subcutaneous tissue). The tumor exhibits irregular margins and heterogeneous internal echogenicity. **(B)** Color Doppler flow imaging of squamous cell carcinoma showing abundant intralesional vascularity with high-velocity blood flow signals. **(C)** HFUS image showing a well-circumscribed, anechoic to hypoechoic round tumor confined predominantly to the dermal layer with smooth margins and homogeneous internal echogenicity. **(D)** Color Doppler imaging of epidermoid cyst demonstrating minimal to absent internal vascularity.

CDFI revealed marked differences in vascularity between benign and malignant tumors (**Table 1**; **Figure 2**). Benign tumors commonly showed no detectable blood flow or low-grade vascularity, whereas malignant tumors demonstrated abundant blood flow, with most tumors classified as Adler grade III (P < 0.001 in both the training and validation cohorts).

### Ultrasound and clinical features by tumor subtype

Among malignant tumors, BCC most commonly presented as a solid, hypoechoic lesion with irregular margins and involvement of the epidermis and dermis, frequently exhibiting heterogeneous echogenicity and moderate-to-marked vascularity. SCC was more frequently associated with surface hyperkeratosis, irregular or creeping morphology, and full-thickness skin involvement extending into the subcutaneous layer. Among benign tumors, epidermoid cysts were consistently anechoic to hypoechoic, well-circumscribed, and confined to the dermis or subcutaneous layer, with posterior acoustic enhancement and absent vascularity. Nevi were predominantly homogeneous, creeping or regular in shape, and avascular or minimally vascular (**Supplementary Table 1**).

### Logistic regression analysis for predicting malignancy

**Figure 3:**
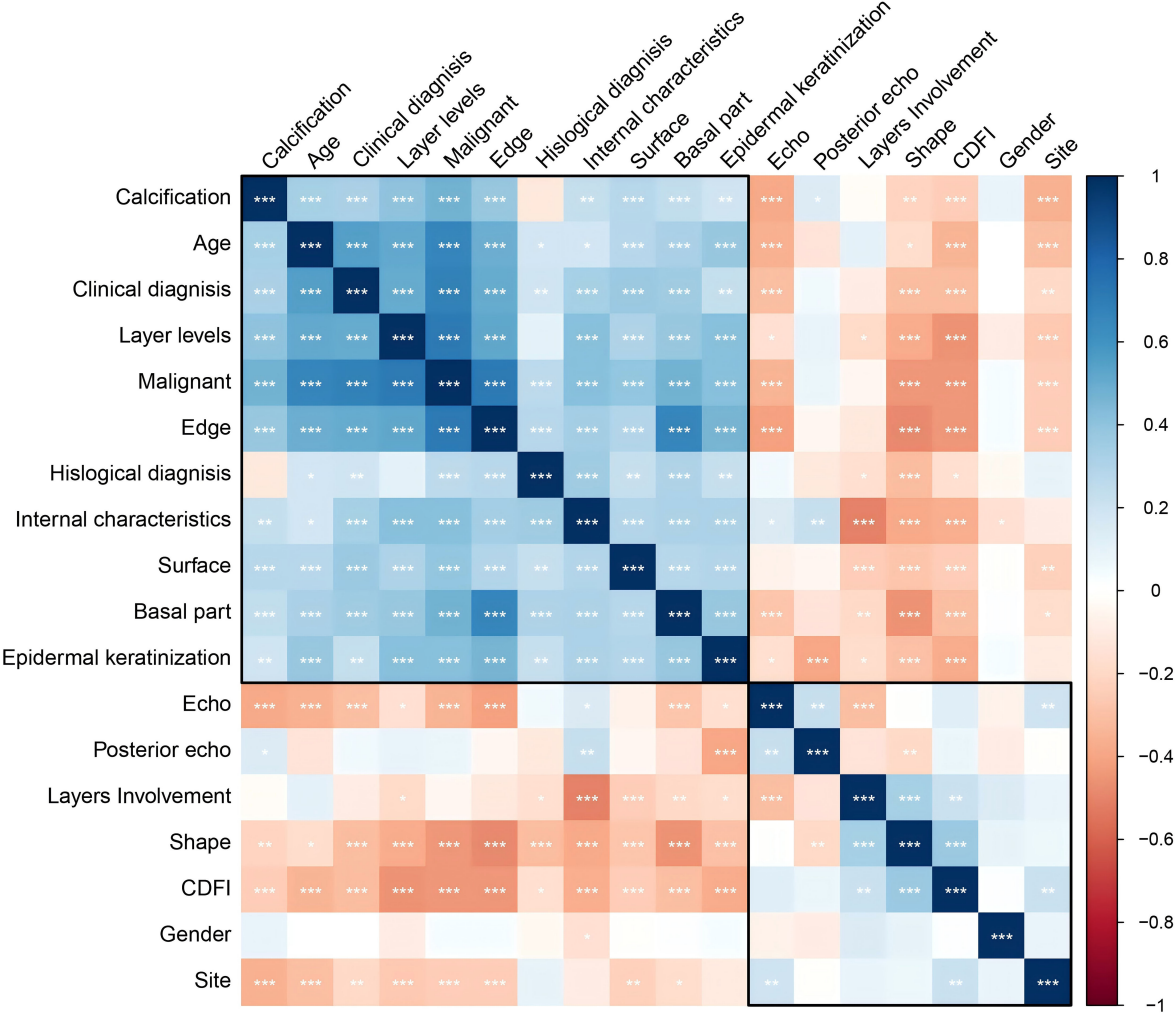
Correlation matrix of clinical and ultrasound features in skin tumor evaluation. Heatmap displaying Spearman’s correlation coefficients between clinical, demographic, and high-frequency ultrasound features in the analysis of benign and malignant skin tumors. The color scale represents correlation strength, ranging from −1 (dark red, strong negative correlation) to +1 (dark blue, strong positive correlation). Asterisks indicate statistical significance levels: *P < 0.05, **P < 0.01, ***P < 0.001.

Multivariate logistic regression analysis was performed to identify independent factors associated with malignant skin tumors (**Figure 3**). The results are summarized in **Table 3**. Among all included variables, age, layer levels, edge, and surface morphology were identified as statistically significant predictors of malignancy. Although heterogeneous echogenicity did not reach conventional statistical significance in multivariate analysis (P = 0.077), it was retained in the nomogram given its borderline P-value, strong univariate association with malignancy, and established pathophysiological rationale. Increasing age was strongly associated with malignancy (OR = 1.01, 95% CI: 1.00-1.01, P < 0.001). Tumors involving a greater number of skin layers showed a significantly higher likelihood of malignancy (OR = 1.19, 95% CI: 1.12-1.26, P < 0.001). Tumors with unclear or irregular edges were more likely to be malignant (OR = 1.36, 95% CI: 1.21-1.54, P < 0.001). Surface characteristics were also significantly associated with malignancy, with raised tumors showing increased odds of malignancy (OR = 1.10, 95% CI: 1.01-1.21, P = 0.031). In contrast, heterogeneous echogenicity was inversely associated with benign tumors and demonstrated a significant relationship with malignancy (OR = 1.09, 95% CI:0.99 - 1.21, P = 0.077).

**Table 3:** Multivariate logistic regression analysis of high-frequency ultrasound features for predicting malignant skin tumors.

Term	OR	Std. error	P-value	95%CI
(Intercept)	0.36	0.25	0.000	0.22-0.58
Age	1.01	0.00	0.000	1.00-1.01
Site	1.04	0.02	0.094	0.99-1.09
Layer levels	1.19	0.03	0.000	1.12-1.26
Shape	0.95	0.03	0.155	0.90-1.02
Edge	1.36	0.06	0.000	1.21-1.54
Basal part	0.95	0.05	0.354	0.86-1.06
Internal characteristics	1.08	0.04	0.052	1.00-1.16
Calcification	1.08	0.05	0.131	0.98-1.19
Surface	1.10	0.05	0.031	1.01-1.21
Epidermal keratinization	1.00	0.03	0.866	0.94-1.05
Echo	1.09	0.05	0.077	0.99-1.21
Posterior echo	1.04	0.03	0.171	0.98-1.11
CDFI	1.01	0.03	0.838	0.95-1.07

CI, Confidence Interval; OR, Odds Ratio; Std. Error, Standard Error.

Variables including tumor site, shape, basal boundary, internal characteristics, calcification, epidermal keratinization, posterior acoustic features, and CDFI grade did not reach statistical significance in the multivariate model (P > 0.05).

### Diagnostic performance and ROC curve analysis

**Figure 4:**
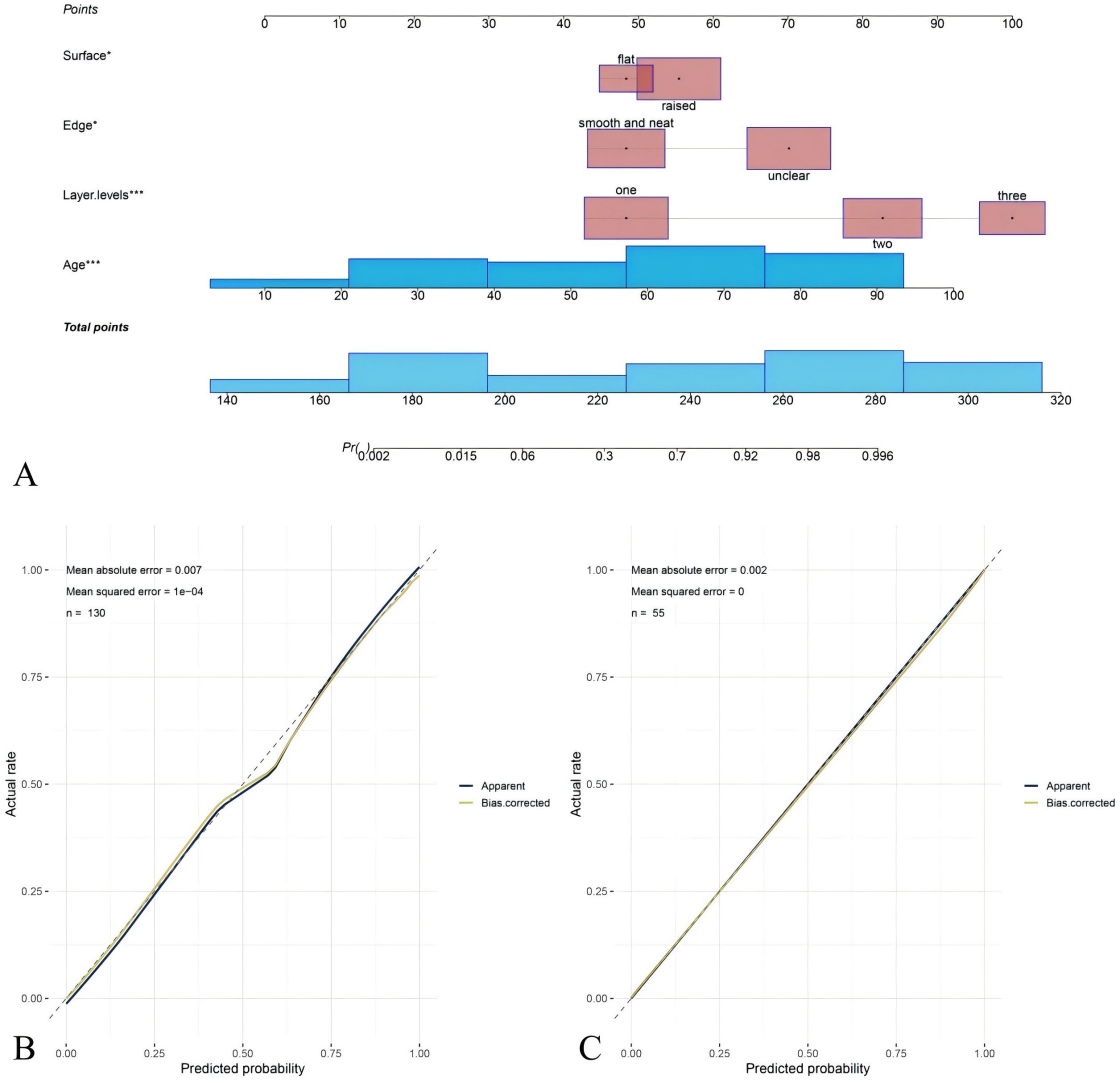
Nomogram for predicting skin tumor malignancy and calibration curve analysis. **(A)** Nomogram integrating independent predictive factors identified by multivariate logistic regression analysis for preoperative prediction of malignant skin tumors. The nomogram incorporates four ultrasound features (surface morphology, edge characteristics, and layer levels) and one clinical variable (age). Each predictor is assigned a point value on the top scale. To use the nomogram, a vertical line is drawn from each variable to the Points axis to determine its contribution. The individual points are summed to obtain the Total points, which corresponds to a predicted probability (Pr) of malignancy on the bottom scale. **(B)** Calibration curve for the nomogram in the training cohort and **(C)** validation cohort.

The nomogram demonstrated excellent discriminative ability in both the training cohort (AUC = 0.98, 95% CI: 0.95-1.00; sensitivity=0.99, specificity=0.96, PPV = 0.97, NPV = 0.98) and the validation cohort (AUC = 0.98, 95% CI: 0.95-1.00; sensitivity=0.97, specificity=0.92, PPV = 0.94, NPV = 0.96), indicating robust generalizability (**Figure 4**; **Table 4**). Among individual predictors, layer involvement and edge characteristics consistently showed the highest single-feature AUCs across both cohorts (training: 0.89 and 0.82; validation: 0.91 and 0.94, respectively), while surface morphology and echogenicity showed lower and more variable performance (AUC range: 0.57-0.72). An AUC of 0.98 indicates near-optimal discrimination between malignant and benign tumors, corresponding to a clinically meaningful ability to correctly stratify the vast majority of patients without the need for invasive biopsy.

**Table 4:** Diagnostic performance of the nomogram and individual predictors in training and validation cohorts.

Cohort	Parameter	AUC	95% CI	SPE	SEN	NPV	PPV
Training	Nomogram	0.98	0.95-1.00	0.96	0.99	0.98	0.97
	Age	0.90	0.84-0.95	0.75	0.90	0.86	0.82
	Layer levels	0.89	0.82-0.95	0.86	0.96	0.94	0.90
	Edge	0.82	0.76-0.89	0.82	0.82	0.78	0.86
	Surface	0.72	0.65-0.8	0.54	0.90	0.82	0.72
	Echo	0.67	0.59-0.75	0.54	0.79	0.67	0.69
Validation	Nomogram	0.98	0.95-1.00	0.92	0.97	0.96	0.94
	Age	0.83	0.71-0.95	0.71	0.97	0.94	0.81
	Layer levels	0.91	0.82-1.00	0.88	0.97	0.95	0.91
	Edge	0.94	0.87-1.00	0.88	1.00	1.00	0.91
	Surface	0.57	0.45-0.7	0.38	0.77	0.56	0.62
	Echo	0.66	0.53-0.78	0.54	0.77	0.65	0.69

AUC, Area Under the Curve; CI, Confidence Interval; SPE, Specificity; SEN, Sensitivity; NPV, Negative Predictive Value; PPV, Positive Predictive Value; ROC, Receiver Operating Characteristic.

### Clinical utility assessment

**Figure 5:**
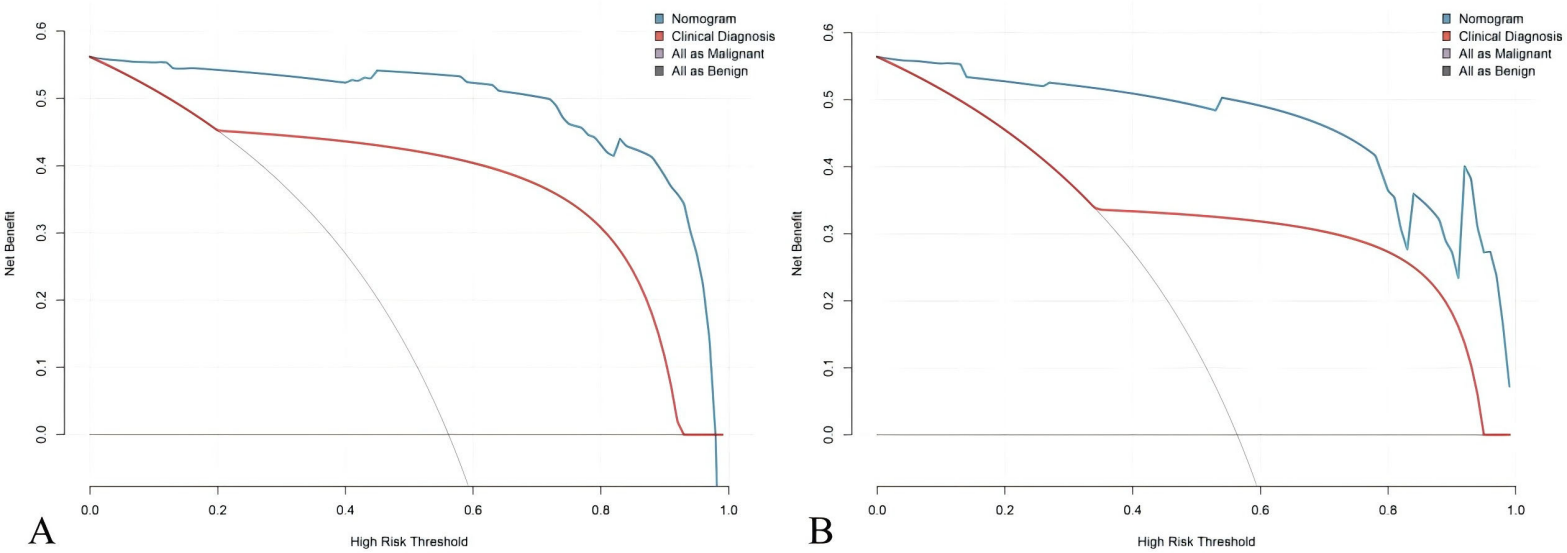
Decision curve analysis (DCA) comparing the clinical utility of the nomogram versus clinical diagnosis. **(A)** DCA in the training cohort and **(B)** validation cohort. The x-axis represents the threshold probability for predicting malignancy, and the y-axis represents the net benefit. The nomogram (blue line) demonstrates superior net benefit compared to clinical diagnosis (red line) across most clinically relevant threshold probabilities. The gray line represents the strategy of treating all patients as malignant, while the black line at zero represents treating all patients as benign. The nomogram maintains positive net benefit across a wide range of threshold probabilities (approximately 0.0-0.95), indicating that using the nomogram to guide clinical decisions would provide greater benefit than either clinical diagnosis alone or the treat-all/treat-none strategies.

The DCA revealed that the malignancy prediction nomogram yielded net benefit gains across both the training and validation datasets (**Figure 5**). The nomogram outperformed default strategies of treating all patients or treating none throughout the clinically relevant range of threshold probabilities.

## Discussion

In this study, the diagnostic value of HFUS in the preoperative differentiation of benign and malignant skin tumors was evaluated. A nomogram integrating clinical and ultrasound features was developed. Results demonstrate that HFUS provides valuable morphologic and structural information. A nomogram prediction model based on HFUS features outperforms clinical assessment alone.

Compared with conventional ultrasound, HFUS offers superior spatial resolution, allowing precise visualization of epidermal, dermal, and subcutaneous structures (12). Previous studies have demonstrated that HFUS can accurately depict tumor margins, depth, and internal architecture, which are critical parameters for preoperative assessment and surgical planning (13, 14). Our findings further confirm that HFUS is highly effective in distinguishing malignant from benign skin tumors based on a combination of morphological and structural characteristics. In our cohorts, malignant tumors were associated with older age, multilayer involvement, irregular shape, unclear or spiculated margins, solid internal composition, heterogeneous echogenicity, and abundant intralesional blood flow. These features are consistent with the biological behavior of malignant tumors, which tend to grow invasively, disrupt normal tissue planes, and induce neovascularization (15, 16).

In this study, association between layer levels and malignancy was shown. Malignant tumors predominantly involved two or three skin layers, whereas benign tumors were usually confined to a single layer, most commonly the subcutaneous tissue. This observation is in accordance with prior reports showing that invasive growth across skin layers is a marker of malignancy (17). Furthermore, tumors with unclear or irregular edges were more likely to be malignant (18). This result reflects infiltrative growth patterns and poor tumor encapsulation. Similar findings have been reported in studies of basal cell carcinoma, squamous cell carcinoma, and melanoma using HFUS (19, 20).

Malignant tumors were more likely to show heterogeneous echogenicity, raised surface contours, and excessive epidermal keratinization. Heterogeneous echogenicity may reflect necrosis, fibrosis, keratin debris, or irregular cellular architecture, all of which are commonly observed in malignant tumors (21). Raised or protruding surfaces and hyperkeratosis are frequently associated with keratinizing tumors such as squamous cell carcinoma and verrucous carcinoma (22, 23). However, some parameters, including basal boundary, internal characteristics, calcification, posterior acoustic features, and CDFI grade, did not remain independently significant in multivariate analysis. This may be due to correlations between variables, overlapping imaging appearances among different tumor types, or limited sample size for certain pathological subgroups.

By integrating age, layer levels, edge characteristics, surface morphology, and echogenicity, we constructed a nomogram that demonstrated good discriminative ability in both training and validation cohorts. DCA demonstrated that the nomogram provided superior net clinical benefit compared with clinical diagnosis alone across a wide range of threshold probabilities. This finding suggests that the nomogram could serve as a practical decision-support tool in clinical settings, helping clinicians identify high-risk tumors, optimize surgical planning, and reduce unnecessary biopsies. Similar predictive models have been proposed in breast, thyroid, and soft tissue tumor imaging, but few studies have focused specifically on skin tumors using HFUS (24, 25). HFUS offers a unique advantage in evaluating tumor thickness and deep extension while remaining noninvasive, real-time, and cost-effective. Our results support the complementary role of HFUS alongside existing dermatologic imaging techniques.

The result of our nomogram in both cohorts compares favorably with previously reported HFUS-based diagnostic studies. Bhatt et al. reported that HFUS correctly identified malignant skin tumors using morphological features alone (9). Qin et al. demonstrated that HFUS could differentiate basal cell carcinoma from common benign pigmented lesions such as seborrheic keratosis with sensitivity and specificity of 90.7% and 84.4% (13). More recently, Song and Chen reported sensitivity and specificity of 94.0% and 93.4% for HFUS-based differentiation of benign from malignant pigmented lesions (11). The superior performance of our nomogram may be attributed to the systematic integration of five independent predictors rather than reliance on individual features. Furthermore, our model was validated in an independent cohort, lending greater credibility to its generalizability than single-cohort analyses.

Several limitations of this study should be acknowledged. First, its retrospective design may introduce selection bias. Second, although the overall sample size was relatively large, some malignant subtypes were underrepresented, which may limit subtype-specific conclusions. Third, external validation in multicenter, prospective cohorts is still required. Finally, while layer involvement was included as a categorical surrogate for tumor depth, a formal quantitative analysis of ultrasonographic tumor thickness and its correlation with histopathological depth of invasion was not performed. Future studies should incorporate depth of invasion as a continuous predictor variable to refine nomogram performance and enhance its applicability to surgical planning. Future studies should focus on prospective multicenter validation, integration of HFUS with advanced techniques such as contrast-enhanced ultrasound and elastography, and the application of artificial intelligence to further improve diagnostic accuracy and reproducibility.

## Conclusion

In conclusion, HFUS is a valuable noninvasive imaging modality for preoperative evaluation of skin tumors, providing detailed morphological and structural information that significantly enhances diagnostic accuracy. The validated nomogram integrating age and key HFUS features demonstrates clinical usefulness in distinguishing malignant from benign tumors and offers superior clinical utility compared to conventional assessment methods.

## Data Availability

The raw data supporting the conclusions of this article will be made available by the authors, without undue reservation.
