# Supplementary material for: Diagnostic value of high-frequency ultrasound in preoperative evaluation of benign and malignant skin tumors
Source: Front Oncol. 2026 Jun 10;16:1819735. doi: 10.3389/fonc.2026.1819735 (PMC13290524; doi:10.3389/fonc.2026.1819735)
Supplement: Supplementary file 1 [file Table1.docx]

**Supplementary Table 1. Clinical and Ultrasound Characteristics of Basal cell carcinoma, Squamous cell carcinoma, Epidermoid cyst, and Nevus**

| Parameters | Basal cell carcinoma | Squamous cell carcinoma | Epidermoid cyst | Nevus | BCC vs EC | BCC vs Nevus | SCC vs EC | SCC vs Nevus |
| --- | --- | --- | --- | --- | --- | --- | --- | --- |
|  | (N=36) | (N=38) | (N=28) | (N=17) |  |  |  |  |
| Group |  |  |  |  |  |  |  |  |
| Malignant | 36 (100%) | 38 (100%) | 0 (0%) | 0 (0%) | <0.001 | <0.001 | <0.001 | <0.001 |
| Benign | 0 (0%) | 0 (0%) | 28 (100%) | 17 (100%) |  |  |  |  |
| Gender |  |  |  |  |  |  |  |  |
| Female | 16 (44.4%) | 20 (52.6%) | 11 (39.3%) | 12 (70.6%) | 0.873 | 0.138 | 0.41 | 0.341 |
| Male | 20 (55.6%) | 18 (47.4%) | 17 (60.7%) | 5 (29.4%) |  |  |  |  |
| Age | 70.6±13.4 | 75.2±15.3 | 45.8±16.2 | 26.7±14.3 | <0.001 | <0.001 | <0.001 | <0.001 |
| Site |  |  |  |  |  |  |  |  |
| Head and Face | 29 (80.6%) | 19 (50.0%) | 9 (32.1%) | 6 (35.3%) | <0.001 | 0.002 | 0.014 | 0.222 |
| Limb | 3 (8.3%) | 9 (23.7%) | 2 (7.1%) | 8 (47.1%) |  |  |  |  |
| Trunk | 4 (11.1%) | 10 (26.3%) | 17 (60.7%) | 3 (17.6%) |  |  |  |  |
| Clinical diagnisis |  |  |  |  |  |  |  |  |
| Benign | 9 (25.0%) | 8 (21.1%) | 27 (96.4%) | 15 (88.2%) | <0.001 | <0.001 | <0.001 | <0.001 |
| Malignant | 27 (75.0%) | 30 (78.9%) | 1 (3.6%) | 2 (11.8%) |  |  |  |  |
| Layers Involvement |  |  |  |  |  |  |  |  |
| Epidermis and dermis | 25 (69.4%) | 22 (57.9%) | 0 (0%) | 4 (23.5%) | <0.001 | <0.001 | <0.001 | <0.001 |
| Epidermis and dermis and subcutaneous | 11 (30.6%) | 16 (42.1%) | 0 (0%) | 0 (0%) |  |  |  |  |
| Subcutaneous | 0 (0%) | 0 (0%) | 28 (100%) | 0 (0%) |  |  |  |  |
| Dermis | 0 (0%) | 0 (0%) | 0 (0%) | 6 (35.3%) |  |  |  |  |
| Epidermis | 0 (0%) | 0 (0%) | 0 (0%) | 7 (41.2%) |  |  |  |  |
| Layer levels |  |  |  |  |  |  |  |  |
| Three | 11 (30.6%) | 16 (42.1%) | 0 (0%) | 0 (0%) | <0.001 | <0.001 | <0.001 | <0.001 |
| Two | 25 (69.4%) | 22 (57.9%) | 0 (0%) | 5 (29.4%) |  |  |  |  |
| One | 0 (0%) | 0 (0%) | 28 (100%) | 12 (70.6%) |  |  |  |  |
| Shape |  |  |  |  |  |  |  |  |
| Creeping | 6 (16.7%) | 8 (21.1%) | 0 (0%) | 10 (58.8%) | <0.001 | 0.001 | <0.001 | <0.001 |
| Irregular | 19 (52.8%) | 25 (65.8%) | 1 (3.6%) | 1 (5.9%) |  |  |  |  |
| Regular | 11 (30.6%) | 5 (13.2%) | 27 (96.4%) | 6 (35.3%) |  |  |  |  |
| Edge |  |  |  |  |  |  |  |  |
| Smooth and neat | 7 (19.4%) | 4 (10.5%) | 25 (89.3%) | 14 (82.4%) | <0.001 | <0.001 | <0.001 | <0.001 |
| Unclear | 29 (80.6%) | 34 (89.5%) | 3 (10.7%) | 3 (17.6%) |  |  |  |  |
| Basal part |  |  |  |  |  |  |  |  |
| Clear | 13 (36.1%) | 10 (26.3%) | 26 (92.9%) | 7 (41.2%) | <0.001 | 0.959 | <0.001 | 0.432 |
| Unclear | 23 (63.9%) | 28 (73.7%) | 2 (7.1%) | 10 (58.8%) |  |  |  |  |
| Internal characteristics |  |  |  |  |  |  |  |  |
| Mixed | 2 (5.6%) | 0 (0%) | 10 (35.7%) | 0 (0%) | <0.001 | 0.827 | <0.001 | 0.005 |
| Solid | 34 (94.4%) | 38 (100%) | 1 (3.6%) | 17 (100%) |  |  |  |  |
| Cystic | 0 (0%) | 0 (0%) | 17 (60.7%) | 0 (0%) |  |  |  |  |
| Calcification |  |  |  |  |  |  |  |  |
| None | 8 (22.2%) | 25 (65.8%) | 26 (92.9%) | 17 (100%) | <0.001 | <0.001 | 0.022 | 0.016 |
| Present | 28 (77.8%) | 13 (34.2%) | 2 (7.1%) | 0 (0%) |  |  |  |  |
| Surface |  |  |  |  |  |  |  |  |
| Flat | 6 (16.7%) | 5 (13.2%) | 18 (64.3%) | 5 (29.4%) | <0.001 | 0.481 | <0.001 | 0.286 |
| Raised | 30 (83.3%) | 33 (86.8%) | 10 (35.7%) | 12 (70.6%) |  |  |  |  |
| Epidermal keratinization |  |  |  |  |  |  |  |  |
| None | 13 (36.1%) | 14 (36.8%) | 28 (100%) | 12 (70.6%) | <0.001 | 0.062 | <0.001 | 0.005 |
| Normal keratinization | 12 (33.3%) | 2 (5.3%) | 0 (0%) | 3 (17.6%) |  |  |  |  |
| Over keratinization | 11 (30.6%) | 22 (57.9%) | 0 (0%) | 2 (11.8%) |  |  |  |  |
| Echo |  |  |  |  |  |  |  |  |
| Non-uniform | 30 (83.3%) | 29 (76.3%) | 24 (85.7%) | 1 (5.9%) | 1 | <0.001 | 0.525 | <0.001 |
| Uniform | 6 (16.7%) | 9 (23.7%) | 4 (14.3%) | 16 (94.1%) |  |  |  |  |
| Posterior echo |  |  |  |  |  |  |  |  |
| Attenuated | 3 (8.3%) | 12 (31.6%) | 0 (0%) | 2 (11.8%) | <0.001 | 0.579 | <0.001 | 0.215 |
| Enhanced | 2 (5.6%) | 1 (2.6%) | 25 (89.3%) | 0 (0%) |  |  |  |  |
| Unchanged | 31 (86.1%) | 25 (65.8%) | 3 (10.7%) | 15 (88.2%) |  |  |  |  |
| CDFI |  |  |  |  |  |  |  |  |
| I | 1 (2.8%) | 2 (5.3%) | 0 (0%) | 3 (17.6%) | <0.001 | <0.001 | <0.001 | <0.001 |
| II | 4 (11.1%) | 0 (0%) | 0 (0%) | 0 (0%) |  |  |  |  |
| III | 31 (86.1%) | 35 (92.1%) | 2 (7.1%) | 1 (5.9%) |  |  |  |  |
| None | 0 (0%) | 1 (2.6%) | 26 (92.9%) | 13 (76.5%) |  |  |  |  |

BCC, Basal cell carcinoma; EC, Epidermoid cyst; SCC, Squamous cell carcinoma
